# A Mosaic *PHEX* Variant in Hypophosphatemic Rickets: Distinguishing Postzygotic Mutation from Sex Chromosome Aneuploidy

**DOI:** 10.1007/s00223-025-01388-4

**Published:** 2025-06-18

**Authors:** Wu Tzen Lim, Marni Nenke, Lesley Rawlings, Amanda Wells, Cassandra Vakulin, Wendy Waters, Sunita De Sousa

**Affiliations:** 1 Adelaide Medical School, University of Adelaide, Adelaide, Australia; 2 Endocrine and Metabolic Unit, Royal Adelaide Hospital, Adelaide, Australia; 3 Diabetes and Endocrine Service, The Queen Elizabeth Hospital, Woodville South, Australia; 4 Department of Genetics & Molecular Pathology, SA Pathology, Adelaide, Australia; 5 Adult Genetics Unit, Royal Adelaide Hospital, Adelaide, Australia

**Keywords:** X-linked hypophosphatemia, Hypophosphatemic rickets, Mosaic, De novo, Postzygotic mutation

## Abstract

Hereditary hypophosphatemic rickets, most commonly caused by X-linked dominant *PHEX* variants, leads to hypophosphatemia and bone mineralization defects. We identified a novel mosaic nonsense variant in the *PHEX* gene on the X chromosome by next-generation sequencing—c.1971C > A, p.(Tyr657X)—–in a man with clinical features of hypophosphatemic rickets. As the variant was only found in 67% of DNA reads, we considered the possibility of sex chromosome aneuploidy (e.g. a 48,XXXY sex chromosome complement with an unaffected X chromosome i.e. variant on 2 of 3 X chromosomes producing a variant allele frequency of approx. 67%) or a postzygotic mutation resulting in the *PHEX* variant in some but not all cells. His mother was clinically unaffected, and he did not have features of Klinefelter’s syndrome, favouring postzygotic mutation over sex chromosome aneuploidy. We excluded sex chromosome aneuploidy through karyotype studies showing a 46,XY status. As the event must therefore be a postzygotic variant to produce the reduced variant allele frequency, his parents are not at risk of having the variant. However, X chromosome postzygotic mutations in men may be inherited by female offspring (depending on the mosaic status of gonadal tissue). The patient’s karyotype result was thus integral in the investigation of disease mechanism and in guiding family genetic counselling.

## Introduction

Familial hypophosphatemic rickets is the most prevalent inherited type of rickets, marked by hypophosphatemia resulting from reduced renal tubular reabsorption of inorganic phosphate and an inadequately low serum level of 1,25-dihydroxyvitamin D. It typically presents with short stature, dental abnormalities, and impaired bone mineralization [1].

Hereditary hypophosphatemic rickets exhibits locus heterogeneity, meaning it can result from variations in multiple genes, which supports the use of gene panel testing for molecular diagnosis. The most common cause is *PHEX* variants, inherited in an X-linked dominant pattern with complete penetrance. In affected individuals, the variant allele fraction should be 100% in males (due to their single X chromosome) and 50% in females (as one of two X chromosomes is affected); deviations from these percentages suggest variant mosaicism.

Using next-generation sequencing, we identified a novel mosaic nonsense variant in the *PHEX* gene on the X chromosome—c.1971C > A, p.(Tyr657X)—in a man with clinical features of hypophosphatemic rickets. The variant was detected in 67% of DNA reads, confirming its postzygotic origin after excluding chromosome aneuploidy.

## Case Presentation

A 56-year-old man presented to the Endocrine Department for review of diffuse vertebral osteosclerosis with smaller foci of sclerosis in the right humerus, scapula, pelvis, sacrum, ribs, left ulna and left tibia on X-ray and computed tomography imaging. This had been identified on imaging performed in the investigation of chronic back pain and elevated alkaline phosphatase (ALP) levels, both of which had been present for at least four years with no clear aetiology identified. He had no history of fractures. He reported losing seven teeth over the preceding few years due to minimal trauma and requiring ten fillings. He also had features suggestive of enthesopathy, affecting both his hands and feet and limiting his mobility.

His past medical history was significant for bilateral genu varum, requiring surgical correction at age 2 years and bilateral high tibial osteotomy at age 44 and 47, and scoliosis attributed to right leg shortening. He also underwent L4-L5/S1 laminectomy in 2015 for severe spinal canal stenosis. Additionally, he had a history of ischaemic heart disease. His current medications were aspirin, ticagrelor, atorvastatin, metoprolol, and glyceryl trinitrate as required. The patient has one son and one daughter with no significant family history of bone disorders.

The patient was referred to our centre in 2022. Biochemistry at that time revealed low phosphate levels, at least since 2022, with phosphate levels ranging from 0.56 to 0.61 [normal range 0.75–1.50 mmol/L]. Historical results prior to this were not available. Ionized calcium levels were normal. Parathyroid hormone (PTH) level was normal at 5.2 [0.87–5.5 mmol/L], while alkaline phosphatase (ALP) levels were mildly elevated, ranging from 131 to 138 [normal range 30–110 mmol/L]. 25-hydroxyvitamin D level was low at 52 [normal, > 60 mmol/L]. 24-h urine phosphate was inappropriately mid-normal range at 30 mmol/24 h [13–55 mmol/L over 24 h], with a creatinine level of 87. Calculated TmP-GFR was low at 0.16 mmol/L, consistent with renal phosphate wasting. Biochemistry is shown in Table 1. He was commenced on oral phosphate replacement and calcitriol which was poorly tolerated.

**Table 1:** Blood and urine tests summary

Blood profile		
	Reference range	Patient’s value
Phosphate	0.75- 1.50 mmol/L	0.56
Ionised Calcium	1.10 -2.20 mmol/L	1.17
Serum creatinine	60-110umol/L	87
Serum alkaline phosphatase	30–110 IU/L	138
Parathyroid hormone	0.8–5.5 pmol/L	5.2
25-hydroxyvitamin D3	> 60 mmol/L	52
Urine profile		
24 urine phosphate	13-55 mmol/L over 24 h	30
Tubular threshold of phosphate per volume unit of glomerular filtration rate (TmPO4/GFR)	0.80 – 1.35 mmol/L	0.16

Investigation for osteosclerosis included PSA, viral hepatitis screen, tryptase, protein electrophoresis, vitamin C, cryoglobulins, and urinary fluoride, all of which were unremarkable. He underwent a whole-body bone scan, which showed no evidence of active Paget’s disease or osteoblastic metastatic disease. His bone mineral density was significant for a T-score at the lumbar spine of + 10, Z-score + 9.9.

He was thought to most likely have a genetic form of hypophosphatemic rickets. An atypical form of osteopetrosis was also considered given his marked osteosclerosis. With the aim of securing a molecular diagnosis, he underwent germline next-generation sequencing (NGS) gene panel testing of genes associated with hereditary hypophosphatemic rickets or osteopetrosis. This revealed a novel mosaic nonsense variant, c.1971C > A, p.( Tyr657X) in exon 20/22 of the *PHEX* gene that is absent from population databases and has not been reported in dbSNP, ClinVar, HGMD or the scientific literature to date. The variant was classified as likely pathogenic (PVS1 and PM2 by American College of Medical Genetics and Genomics variant classification criteria).

Notably, the *PHEX* variant had an allele frequency of only 67% rather than 100% as expected for X chromosome variants in male patients. We concluded that he must have either the unlikely event of 48,XXXY sex chromosome aneuploidy with unaffected X chromosomes (i.e. variant on 2 of 3 X chromosomes producing a variant allele frequency of approx. 67%) or a postzygotic mutation resulting in the *PHEX* variant in some but not all cells. Given his mother was clinically unaffected, and he did not have features of Klinefelter’s syndrome, postzygotic mutation was favoured over sex chromosome aneuploidy as the explanation for his mosaicism. As this distinction would guide family genetic counselling, we proceeded to perform a karyotype. The result was 46,XY, thereby excluding sex chromosome aneuploidy.

The final diagnosis was X-linked hypophosphatemic rickets due to a novel postzygotic mosaic *PHEX* variant.

The patient was switched from calcitriol and phosphate replacement to burosumab 90 mg 4 weekly, accessed via the Australian Pharmaceutical Benefits Scheme (PBS) using the indication of X-linked hypophosphatemia with a confirmed *PHEX* pathogenic variant.

One month after his first dose of burosumab, he was found to be hyperphosphatemic with phosphate level of 1.65 [0.75–1.50 mmol/L]. Following dosing guidelines, burosumab was withheld until phosphate normalised, at which time the dose was reduced to 40 mg monthly. Treatment has been well tolerated, although joint pain and stiffness remain.

## Discussion

Familial hypophosphatemic rickets is the most common inherited form of rickets and is characterised by hypophosphatemia due to decreased renal tubular reabsorption of inorganic phosphate and an inappropriately low serum concentration of 1,25-dihydroxyvitamin D [1]. The condition usually manifests as short stature, dental anomalies and defective bone mineralisation.

Hereditary hypophosphatemic rickets is most commonly the X-linked dominant form due to variants in the *PHEX* gene located at chromosome Xp22.1-p22.2 [2], but other inheritance patterns exist [3–5]. Autosomal dominant hypophosphatemic rickets is due to FGF23 variants, while autosomal recessive hypophosphatemic rickets is due to biallelic variants in DMP1, ENPP1, FAM20C or SLC34A3. X-linked recessive hypophosphatemic rickets is due to CLCN5 variants.

In our patient, given his negative family history, we suspected that he might have a de novo variant in *PHEX* or possibly one of the rarer recessive forms of hypophosphatemic rickets. Gene panel testing was undertaken to evaluate these different possibilities. Upon finding the *PHEX* variant, we suspected that this would most likely be de novo given that the patient’s mother was clinically unaffected and that the penetrance of XLH in heterozygous females is usually high [6, 7], although his mother might have been clinically unaffected due to her own mosaicism. A de novo variant refers to a genetic alteration that arises for the first time in an individual, rather than being inherited from either parent. It occurs either in a parental germ cell (egg or sperm) or in the fertilized egg during early embryonic development (postzygotic variant). Once present, postzygotic variants are passed on to daughter cells, whilst the cells upstream of the postzygotic mutation will continue to multiply without the variant. [6, 7]. Through subsequent karyotyping confirming 46,XY status (i.e. a single X chromosome per cell) in our patient, we ultimately concluded that this must be a postzygotic variant, which is by definition a de novo event.

To our knowledge, there have been just fifteenth published case reports of male mosaicism for *PHEX* variants [1, 8–17], as shown in Table 2. Sex chromosome aneuploidy was excluded in every case in which this was examined, but this alternative explanation for a reduced variant allele fraction remains a theoretical possibility. This distinction is important because if sex chromosome aneuploidy were present, then this would mean that the X chromosome/s inherited from the mother might be the source of the *PHEX* variant, thus the mother and her relatives would be at risk for the condition. It is also important to note that whilst parental disease is ruled out in postzygotic variants, any daughters (obligatory recipients of an X chromosome from their father) of affected males may inherit one of the X chromosomes containing the *PHEX* variant and therefore inherit the condition. The chance of this occurring will depend on the degree of mosaicism at the level of the testes. Sons, by contrast, are obligatory recipients of their father’s Y chromosome and hence will not inherit the *PHEX* variant or XLH. Family counselling was undertaken with consideration of all these aspects.

**Table 2:** Case of male mosaics for PHEX variants

Article	Cases of mosaic PHEX	Patient’s age	Country of Publication	Presentation	Hypophosphataemia	Short stature	Skeletal abnormalities	Serum alkaline phosphatase level (IU/L) (reference range: 53–128 IU/L)	Family History	Karyotype	*PHEX* Gene mutation	Variant allele frequency (%)
Goji K et al. 2006 [1]	1.	35-year-old	Japan	Had genetic testing as elder daughter presented with XLH while 2nd daughter was normal phenotypically Diagnosed with hypophosphatemia rickets/ osteomalacia at age 2	Y	Y	Y	215	Proband’s daughter	46, XY	Splicing variant c.1669C > T (p.Arg567Ter)	60
Clausmeyer s, et al. (2009) [8]	2.	10-month-old	Germany	Bowing of legs, growth retardation, bone pain	Y	Y	Y	NR	N	46, XY	c.1605_1607delGAC (c.T536del)	NR
Weng C, et al. (2016) [9]	3.	4.5-year-old	China	gait abnormalities and bone pain at 2-year-old	Y	Y	Y	616	Nil	46, XY	Novel Splicing variant C1769-1G > A in intron 17 deletion of 4 bp in exon 18 led to a frameshift mutation, p.Gly590GlufsTer28, resulting in a premature stop codon	42
Yang M, et al. (2018) [10]	4.	26-year-old	South Korea	Incidental finding of bow legs at 3 years old Waddling gait at 13 months	Y	Y	Y	99	Nil	NR	Nonsense mutation c.589C > T p.Gln197Ter in exon 5	38
Zhang C, et al. (2019) [11]	5.	10-year-old	China	NR	Y	N	Y	NR	NR	NR	splice site mutation c.1645 + 4 A > G	NR
	6.	3.5-year-old	China	NR	Y	N	Y	NR	NR	NR	splice site mutation c.933 + 1 G > A	NR
Lin Y, et al. (2020) [12]	7.	NR	China	NR	Y	Y	Y	618	N	46, XY	Missense mutation c.1601C > T(p.P534L)	NR
	8.	NR	China	NR	Y	N	Y	1396	N	46, XY	Nonsense mutation c.2064T > A(p.Y688*)	NR
	9.	NR	China	NR	Y	Y	Y	434	N	46, XY	Missense mutation c.230G > C(p.C77S)	NR
	10.	NR	China	NR	Y	N	Y	455	N	46, XY	Nonsense mutation c.2104C > T(p.R702*)	NR
Broseta JJ, et al. (2020) [13]	11.	2-year-8 month-old	Spain	Bilateral genu varum since 9-month-old	Y	NR	Y	659	N	46, XY	c.2182C > T(p.Q728*)	NR
Asano S, et al. (2021) [14]	12.	61-year-old	Japan	coxodynia Noted to have bow legs, short stature and scoliosis Diagnosed with rickets at age of 5 and had multiple dental caries	Y	Y	Y	373	Nil	NR	in-frame deletion NM_000444.6:c.671_685del p.Gln224_Ser228del	21
Lin Y, et al. (2020) [15]	13.	35-year-old	China	Had genetic testing as daughter was diagnosed with XLH	N	N	N	75	Proband’s daughter	NR	c.1666C > T(p.Q556*	26.67
Terracciano A, et al. (2023) [16]	14.	34th month old	Italy	Growth retardation, abnormal gait and skeletal changes	Y	Y	Y	902	Nil	NR	Novel nonsense variant c.2176G > T (p.Glu726Ter) in exon 22	47
Ma X, et al. (2024) [17]	15.	7-year-old	China	diagnosed with hypophosphatemia at age of 11 months old and skeletal abnormalities	Y	NR	Y	549	Nil	NR	mosaic deletions in exons 6 through 12 of the PHEX gene in the patient	Not reported
Present case	16.	56-year-old	Australia	diagnosed with rickets at age of 2 Has bone changes and multiple teeth losses	Y	Y	Y	138	Nil	46 XY	Mosaic c.1971C > A, p.(Tyr657X)	67

NR: not reported, Y: Yes, N: No

Noting that XLH is primarily due to defective bone mineralisation, the osteosclerosis observed in our patient seems paradoxical. This phenomenon could be multifactorial. Most patients present in childhood, and timely treatment can reduce morbidity. However, adults with XLH are known to develop numerous complications including endochondral and membranous ossification [18]. Our patient had presumably suffered untreated hypophosphatemia for 56 years, likely resulting in complex local pathology in the bone tissue. There are some data available on the biomechanical properties of bone in prolonged untreated XLH. One study comparing bone biopsies from two treated and two non-treated adult patients revealed significant differences in bone material properties whereby untreated XLH bone is not simply undermineralised, but contains a heterogeneous spread of both highly mineralised bone matrix and large amounts of unmineralized osteoid. There was a doubling in trabecular bone volume and trabecularisation of cortical bone [19]. We speculate that the persistent state of osteomalacia in our patient led to increasing heterogeneity of the bone microarchitecture, appearing as sclerotic change. Another explanation (mechanostat theory) may be that the osteocytes in XLH bone will overestimate the mechanical load through the soft osteoid, resulting in localised modelling and/or remodelling to increase bone strength [20–22]. Heterotopic ossification (abnormal formation of bone in soft tissues outside of skeleton) of tendon insertion sites may further contribute to the sclerotic appearance [23–25]. 

The use of burosumab in XLH has been more extensively studied in children compared with adults. Nevertheless, genetic testing is still a valuable tool for confirming a diagnosis of XLH in adults suspected of having the condition as recent studies have demonstrated that burosumab is an effective treatment for adults with XLH, showing significant improvements in phosphate homeostasis, patient-reported outcomes and physical function [26–28].

In conclusion, we have identified a novel mosaic nonsense variant in the *PHEX* gene on the X chromosome—c.1971C > A, p.(Tyr657X)—due to apparent postzygotic mutation in a man with the clinical syndrome of hypophosphatemic rickets. The case illustrates the importance of karyotyping to confirm postzygotic mutation and guide reproductive counselling and cascade testing.

## Data Availability

All data generated or analysed during this study are included in this article. Further enquiries can be directed to the corresponding author.
